# ASO Author Reflections: Registry-Based Analysis of Radiation-Associated Angiosarcoma of the Breast Provides Unbiased Insights into this Disease

**DOI:** 10.1245/s10434-019-08132-x

**Published:** 2019-12-12

**Authors:** Samuli H. Salminen, Carl P. Blomqvist

**Affiliations:** 1 Comprehensive Cancer Center, Helsinki University Hospital (HUH), Helsinki, Finland; 2 University of Helsinki, Helsinki, Finland

## Past

Radiation-associated angiosarcoma of the breast (RAASB) is increasingly diagnosed in breast cancer survivors.^1^ Previous research has highlighted the importance of radical surgery in the management of this disease.^2^ Unfortunately, most published research was performed in single centers, possibly hampering the interpretation of results. A recent registry-based analysis from The Netherlands reported a 5-year overall survival rate of 40.5%.^3^

## Present

We performed a comprehensive analysis of RAASB patients identified from the database of the Finnish Cancer Registry over 25 years.^4^ Thus, our study provides a unique perspective of RAASB treatment and prognosis in a nationwide population with complete follow-up of all patients. We discovered that most RAASB patients are eligible for radical surgery, and therefore possibly curative treatment, emphasizing the importance of awareness of this disease in the rapidly growing population of breast cancer survivors. Forty-five percent of patients later had a local recurrence, and planned lateral surgical margin was associated with improved survival, therefore underlining the impact of radical surgery. Furthermore, locally recurring disease has a tendency to progress into a metastatic disease, further impairing prognosis.

## Future

The evidence from this and previous studies suggests that the primary treatment of a localized RAASB is radical surgery.^2^^–^4 However, our study did not bring further clarity into what surgical margins are needed for local control, due to the retrospective nature of this rare disease and the lack of comprehensive information for all patients.

Regrettably, a number of RAASB patients later progress into metastatic disease.^4^ Due to the rarity of RAASB, few patients are included in prospective trials of medical treatment. Future work could build on the growing knowledge on the molecular biology basis of RAASB to perform prospective studies in the metastatic setting.
